# Google Matrix Analysis of DNA Sequences

**DOI:** 10.1371/journal.pone.0061519

**Published:** 2013-05-09

**Authors:** Vivek Kandiah, Dima L. Shepelyansky

**Affiliations:** Laboratoire de Physique Théorique du CNRS, IRSAMC, Université de Toulouse, UPS, Toulouse, France; The Scripps Research Institute, United States of America

## Abstract

For DNA sequences of various species we construct the Google matrix 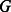 of Markov transitions between nearby words composed of several letters. The statistical distribution of matrix elements of this matrix is shown to be described by a power law with the exponent being close to those of outgoing links in such scale-free networks as the World Wide Web (WWW). At the same time the sum of ingoing matrix elements is characterized by the exponent being significantly larger than those typical for WWW networks. This results in a slow algebraic decay of the PageRank probability determined by the distribution of ingoing elements. The spectrum of 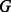 is characterized by a large gap leading to a rapid relaxation process on the DNA sequence networks. We introduce the PageRank proximity correlator between different species which determines their statistical similarity from the view point of Markov chains. The properties of other eigenstates of the Google matrix are also discussed. Our results establish scale-free features of DNA sequence networks showing their similarities and distinctions with the WWW and linguistic networks.

## Introduction

The theory of Markov chains [1] finds impressive modern applications to information retrieval and ranking of directed networks including the World Wide Web (WWW) where the number of nodes is now counted by tens of billions. The PageRank algorithm (PRA) [2] uses the concept of the Google matrix 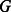 and allows to rank all WWW nodes in an efficient way. This algorithm is a fundamental element of the Google search engine used by a majority of Internet users. A detailed description of this method and basic properties of the Google matrix can be found e.g. in [3], [4].

The Google matrix belongs to the class of Perron-Frobenius operators naturally appearing in dynamical systems (see e.g. [5]). Using the Ulam method [6] a discrete approximant of Perron-Frobenius operator can be constructed for simple dynamical maps following only one trajectory in a chaotic component [7] or using many independent trajectories counting their probability transitions between phase space cells [8], [9], [10]. The studies of Google matrix of such directed Ulam networks provides an interesting and detailed analysis of dynamical properties of maps with a complex chaotic dynamics [7], [8], [9], [10].

In this work we use the Google matrix approach to study the statistical properties of DNA sequences of the species: Homo sapiens (HS, human), Canis familiaris (CF, dog), Loxodonta africana (LA, elephant), Bos Taurus (bull, BT), Danio rerio (DR, zebrafish), taken from the publicly available database [11]. The analysis of Poincaré recurrences in these DNA sequences [12] shows their similarities with the statistical properties of recurrences for dynamical trajectories in the Chirikov standard map and other symplectic maps [7]. Indeed, a DNA sequence can be viewed as a long symbolic trajectory and hence, the Google matrix, constructed from it, highlights the statistical features of DNA from a new viewpoint.

An important step in the statistical analysis of DNA sequences was done in [13] applying methods of statistical linguistics and determining the frequency of various words composed of up to 7 letters. A first order Markovian models have been also proposed and briefly discussed in this work. Here we show that the Google matrix analysis provides a natural extension of this approach. Thus the PageRank eigenvector gives the frequency appearance of words of given length. The spectrum and eigenstates of 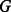 characterize the relaxation processes of different modes in the Markov process generated by a symbolic DNA sequence. We show that the comparison of word ranks of different species allows to identify proximity between species.

At present the investigations of statistical properties of DNA sequences are actively developed by various bioinformatic groups (see e.g. [14], [15], [16], [17], [18]). The development of various methods of statistical analysis of DNA sequences become now of great importance due to a rapid growth of collected genomic data. We hope that the Google matrix approach, which already demonstrated its efficiency for enormously large networks [2], [3], will find useful applications for analysis of genomic data sets.

## Results

### Construction of Google matrix from DNA sequence

From [11] we collected DNA sequences of HS represented as a single string of length 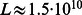 base pairs (bp) corresponding to 5 individuals. Similar data are obtained for BT (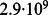 bp), CF (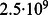 bp), LA (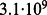 bp), DR (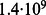 bp). For HS, CF, LA, DR the statistical properties of Poincaré recurrences in these sequences are analyzed in [12]. All strings are composed of 4 letters 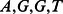 and undetermined letter 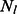. The strings can be found at the web page [19].

For a given sequence we fix the words 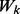 of 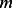 letters length corresponding to the number of states 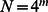. We consider that there is a transition from a state 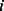 to state 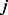 inside this basis 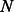 when we move along the string from left to right going from a word 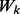 to a next word 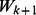. This transition adds one unit in the transition matrix element 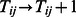. The words with letter 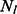 are omitted, the transitions are counted only between nearby words not separated by words with 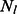. There are approximately 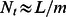 such transitions for the whole length 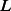 since the fraction of undetermined letters 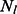 is small. Thus we have 
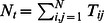
. The Markov matrix of transitions 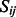 is obtained by normalizing matrix elements in such a way that their sum in each column is equal to unity: 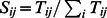. If there are columns with all zero elements (dangling nodes) then zeros of such columns are replaced by 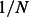. Such a procedure corresponds to one used for the construction of Google matrix of the WWW [2], [3]. Then the Google matrix of DNA sequence is written as
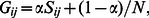
(1)where 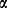 is the damping factor for which the Google search uses usually the value 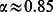 [3]. The matrix 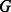 belongs to the class of Perron-Frobenius operators. It has the largest eigenvalue 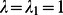 with all other eigenvalues 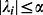. For WWW usually there are isolated subspaces so that at 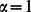 there are many degenerate 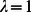 eigenvalues [4] so that the damping factor allows to eliminate this degeneracy creating a gap between 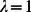 and all other eigenvalues. For our DNA Google matrices we find that there is already a significant spectral gap naturally present. In this case the PageRank vector is not sensitive to the damping factor being in the range 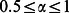 (other eigenvectors are independent of 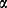 [3], [4], [9]). Due to that in the following we present all results at the value 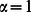.

The spectrum 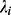 and right eigenstates 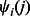 are determined by the equation
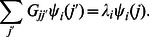
(2)The PageRank eigenvector 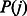 at 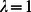 has positive or zero elements which can be interpreted as a probability to find a random surfer on a given site 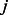 with the total probability normalized to unity 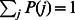. Thus, all sites can be ordered in a decreasing order of probability 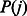 that gives us the PageRank order index 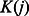 with most frequent sites at low values of 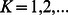.

**Figure 1:**
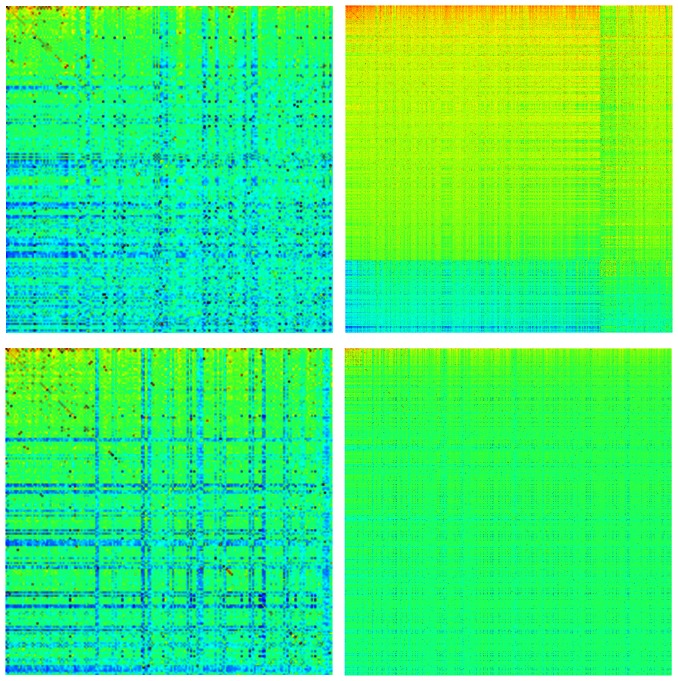
DNA Google matrix of Homo sapiens (HS) constructed for words of 5-letters (top) and 6-letters (bottom) length. Matrix elements 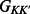 are shown in the basis of PageRank index 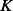 (and 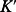). Here, 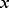 and 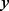 axes show 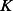 and 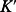 within the range 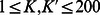 (left) and 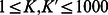 (right). The element 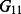 at 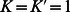 is placed at top left corner. Color marks the amplitude of matrix elements changing from blue for minimum zero value to red at maximum value.

It is useful to consider the density of matrix elements 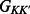 in the PagePank indexes 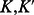 similar to the presentation used in [20], [21] for networks of Wikipedia, UK universities, Linux Kernel and Twitter. The image of the DNA Google matrix of HS is shown in Fig. 1 for words of 5 and 6 letters. We see that almost all matrix is full that is drastically different from the WWW and other networks considered in [20] where the matrix 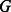 is very sparse. Thus the DNA Google matrix is more similar to the case of Twitter which is characterized by a strong connectivity of top PageRank nodes [21].

**Figure 2:**
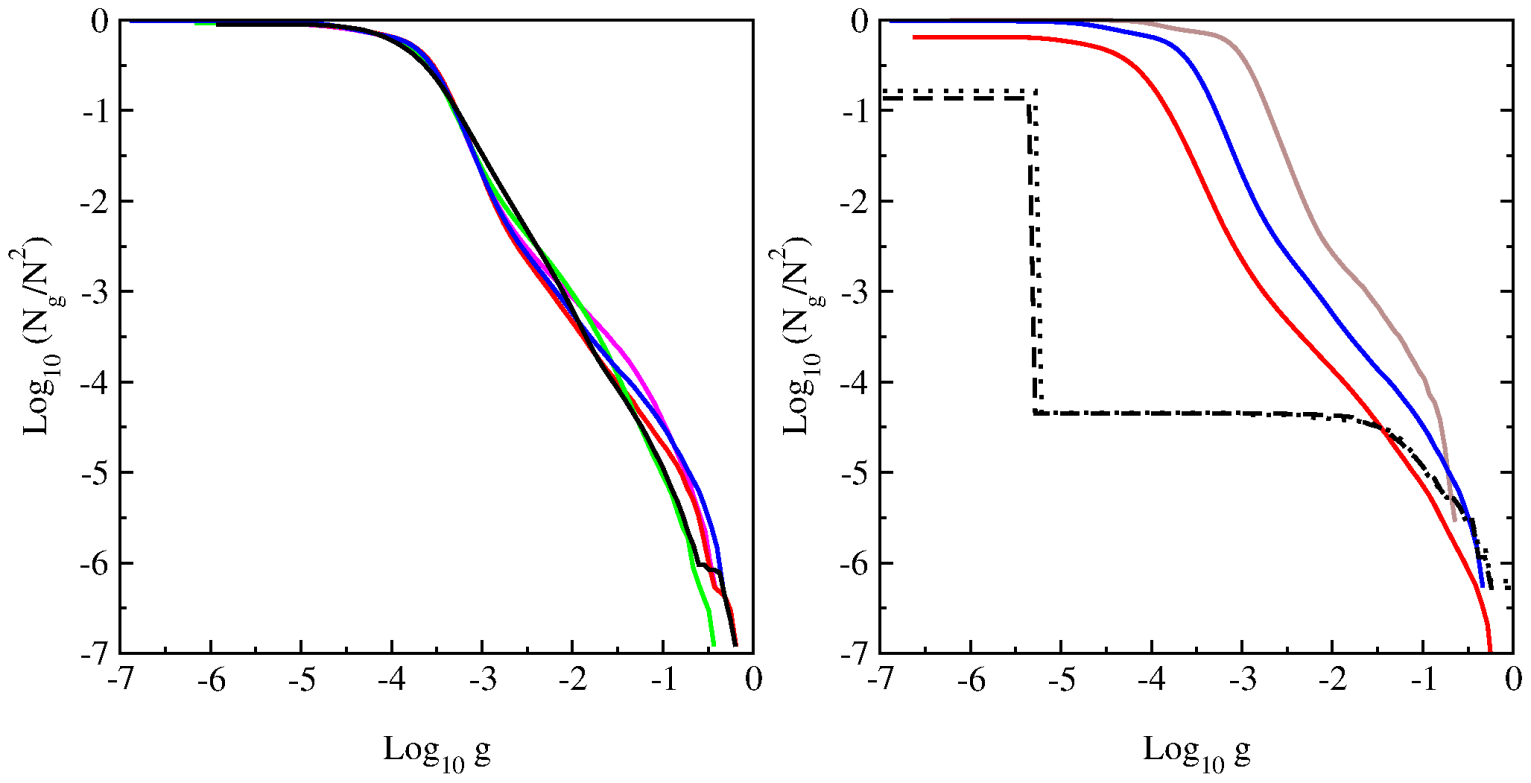
Integrated fraction 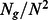 of Google matrix elements with 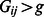 as a function of 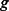. *Left panel :* Various species with 6-letters word length: bull BT (magenta), dog CF (red), elephant LA (green), Homo sapiens HS (blue) and zebrafish DR(black). *Right panel :* Data for HS sequence with words of length 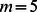 (brown), 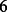 (blue), 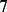 (red). For comparison black dashed and dotted curves show the same distribution for the WWW networks of Universities of Cambridge and Oxford in 2006 respectively.

It is interesting to analyze the statistical properties of matrix elements 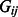. Their integrated distribution is shown in Fig. 2. Here 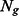 is the number of matrix elements of the matrix 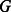 with values 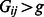. The data show that the number of nonzero matrix elements 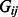 is very close to 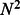. The main fraction of elements has values 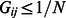 (some elements 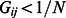 since for certain 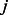 there are many transitions to some node 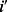 with 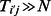 and e.g. only one transition to other 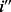 with 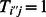). At the same time there are also transition elements 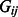 with large values whose fraction decays in an algebraic law 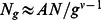 with some constant 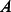 and an exponent 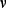. The fit of numerical data in the range 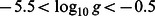 of algebraic decay gives for 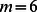: 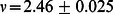 (BT), 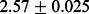 (CF), 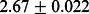 (LA), 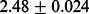 (HS), 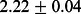 (DR). For HS case we find 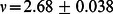 at 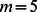 and 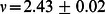 at 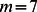 with the average 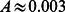 for 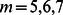. There are visible oscillations in the algebraic decay of 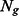 with 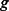 but in global we see that on average all species are well described by a universal decay law with the exponent 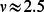. For comparison we also show the distribution 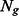 for the WWW networks of University of Cambridge and Oxford in year 2006 (data from [4], [20]). In these networks we have 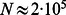 and on average 10 links per node. We see that in these cases the distribution 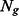 has a very short range in which the decay is at least approximately algebraic (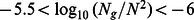). In contrast to that for the DNA sequences we have a large range of algebraic decay.

Since in each column we have the sum of all elements equal to unity we can say that the differential fraction 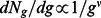 gives the distribution of outgoing matrix elements which is similar to the distribution of outgoing links extensively studied for the WWW networks [3], [23], [24], [25]. Indeed, for the WWW networks all links in a column are considered to have the same weight so that these matrix elements are given by an inverse number of outgoing links [3]. Usually the distribution of outgoing links follows a power law decay with an exponent 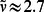 even if it is known that this exponent is much more fluctuating compared to the case of ingoing links. Thus we establish that the distribution of DNA matrix elements is similar to the distribution of outgoing links in the WWW networks with 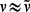. We note that for the distribution of outgoing links of Cambridge and Oxford networks the fit of numerical data gives the exponents 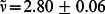 (Cambridge) and 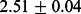 (Oxford).

**Figure 3:**
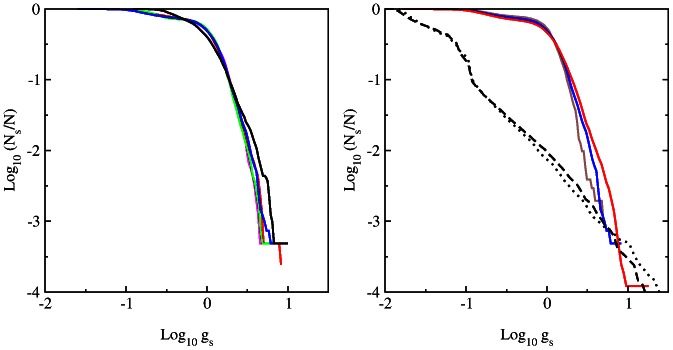
Integrated fraction 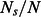 of sum of ingoing matrix elements with 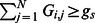. Left and right panels show the same cases as in Fig. 2 in same colors. The dashed and dotted curves are shifted in 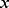-axis by one unit left to fit the figure scale.

It is known that on average the probability of PageRank vector is proportional to the number of ingoing links [3]. This relation is established for scale-free networks with an algebraic distribution of links when the average number of links per node is about 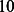 to 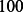 that is usually the case for WWW, Twitter and Wikipedia networks [4], [20], [21], [22], [23], [24], [25]. Thus in such a case the matrix 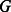 is very sparse. For DNA we find an opposite situation where the Google matrix is almost full and zero matrix elements are practically absent. In such a case an analogue of number of ingoing links is the sum of ingoing matrix elements 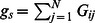. The integrated distribution of ingoing matrix elements with the dependence of 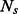 on 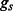 is shown in Fig. 3. Here 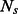 is defined as the number of nodes with the sum of ingoing matrix elements being larger than 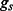. A significant part of this dependence, corresponding to large values of 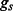 and determining the PageRank probability decay, is well described by a power law 
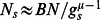
. The fit of data at 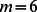 gives 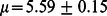 (BT), 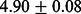 (CF), 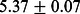 (LA), 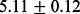 (HS), 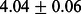 (DR). For HS case at 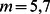 we find respectively 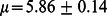 and 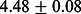. For 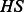 and other species we have an average 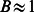.

Usually for ingoing links distribution of WWW and other networks one finds the exponent 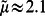 [23], [24], [25]. This value of 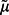 is expected to be the same as the exponent for ingoing matrix elements of matrix 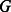. Indeed, for the ingoing matrix elements of Cambridge and Oxford networks we find respectively the exponents 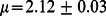 and 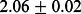 (see curves in Fig. 3). For ingoing links distribution of Cambridge and Oxford networks we obtain respectively 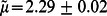 and 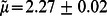 which are close to the usual WWW value 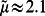. Thus we can say that for the WWW type networks we have 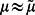. In contrast the exponent 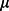 for DNA Google matrix elements gets significantly larger value 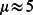. This feature marks a significant difference between DNA and WWW networks.

For DNA we see that there is a certain curvature in addition to a linear decay in log-log scale. From one side, all species are close to a unique universal decay curve which describes the distribution of ingoing matrix elements 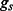 (there is a more pronounced deviation for DR which does not belong to mammalian species). However, from other side we see visible differences between distributions of various species (e.g. non mammalian DR case has the largest deviation from others mammalian species). We will discuss the links between 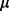 and the exponent 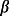 of PageRank algebraic decay 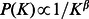 in next sections.

### Spectrum of DNA Google matrix

**Figure 4:**
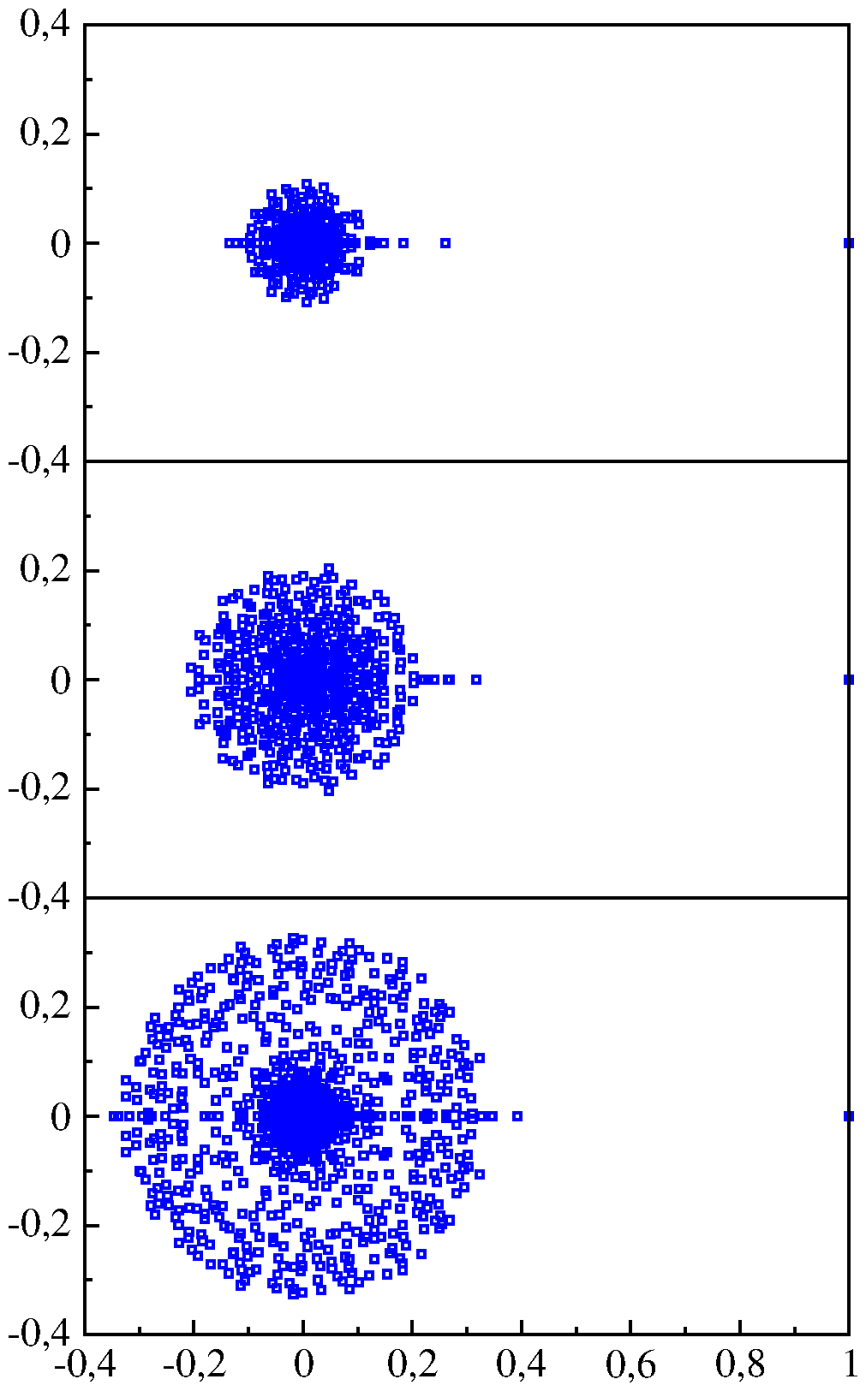
Spectrum of eigenvalues in the complex plane 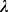 for DNA Google matrix of Homo sapiens (HS) shown for words of 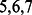 letters (from top to bottom).

**Figure 5:**
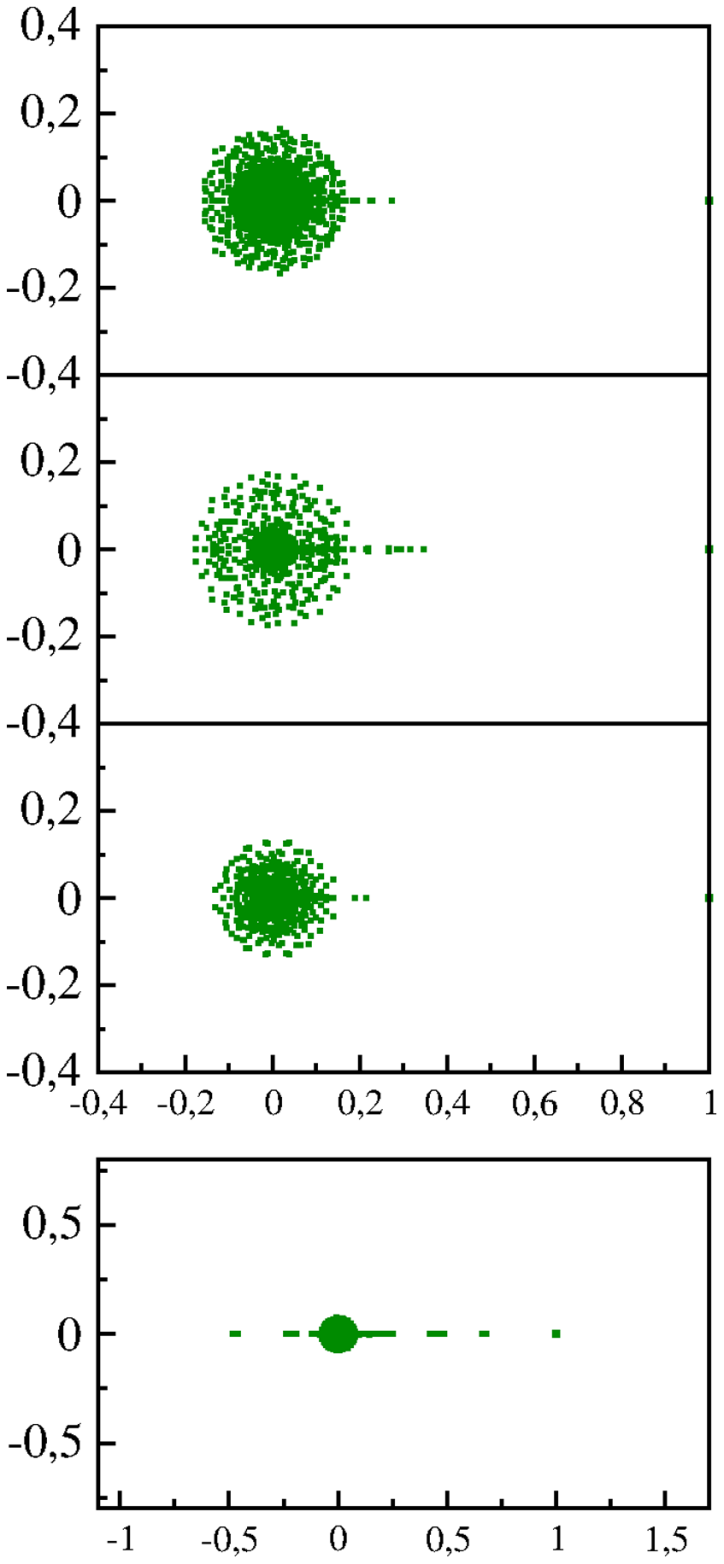
Spectrum of eigenvalues in the complex plane 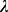 for DNA Google matrix of of bull BT, dog CF, elephant LA, zebrafish DR shown for words of 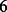 letters (from top to bottom).

The spectrum of eigenstates of DNA Google matrix 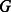 of 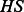 is shown in Fig. 4 for words of 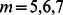 letters and matrix sizes 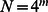. The spectra for DNA sequences of bull BT, dog CF, elephant LA and zebrafish DR are shown in Fig. 5 for words of 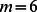 letters. The spectra and eigenstates are obtained by direct numerical diagonalization of matrix 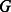 using LAPACK standard code.

In all cases the spectrum has a large gap which separates eigenvalue 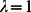 and all other eigenvalues with 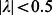 (only for non mammalian DR case we have a small group of eigenvalues within 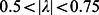). This is drastically different from the spectrum of WWW and other type networks which usually have no gap in the vicinity of 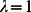 (see e.g. [4], [21], [22]). In a certain sense the DNA 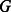 spectrum is similar to the spectrum of randomized WWW networks and the spectrum of 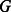 of the Albert-Baraási network model discussed in [26], but the properties of the PageRank vector are rather different as we will see below.

Visually the spectrum is mostly similar between HS and CF having approximately the same radius of circular cloud 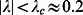. For DR this radius is the smallest with 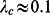. Thus the spectrum of 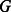 indicates the difference between mammalian and non mammalian sequences. For HS the increase of the word length 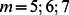 leads to an increase of 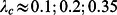. For 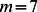 the number of nonzero matrix elements 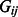 is close to 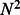 and thus on average we have only about 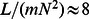 transitions per each element. This determines an approximate limit of reliable statistical computation of matrix elements 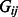 for available HS sequence length 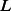. For HS at 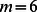 we verified that two halves of the whole sequence 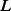 still give practically the same spectrum with a relative accuracy of 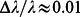 for eigenvalues in the main part of the cloud at 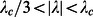. This means that the spectrum presented in Figs 4,5 is statistically stable at the values of 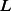 used in this work.

We also constructed the Google matrix 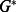 by inverting the direction of transitions 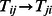 and then normalizing sum of all elements in each column to unity. This procedure is also equivalent to moving along the sequence, from word to word, not from left to right but from right to left. We note that for WWW and other networks such a matrix with inverted direction of links was used to obtain the CheiRank vector (which is the PageRank vector of matrix 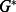). Due to the inversion of links the CheiRank vector highlights very communicative nodes [4], [20], [21], [22]. In our case the spectrum of 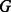 and 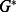 are identical. As a result the probability distributions of PageRank and CheiRank vectors are the same. This is due to some kind of detailed balance principle: we count only transitions between nearby words in a DNA sequence and the direction of displacement along the sequence does not affect the average transition probabilities so that 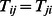 (up to statistical fluctuations). In a certain sense this situation is similar to the case of Ulam networks in symplectic maps where the conservation of phase space area leads to the same properties of 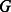 and 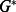 [7], [10].

We tried to test if a random matrix model can reproduce the distribution of eigenvalues in 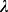 plane. With this aim we generated random matrix elements 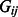 with exactly the same distribution 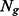 as for HS case at 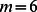 (see Fig. 2). However, in this random model we found all eigenvalues homogeneously distributed in the radius 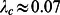 being significantly smaller compared to the real data. Also in this case the PageRank probability 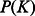 changes only by 30% in the whole range 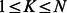 being absolutely different from the real data (see next section). Thus the construction of random matrix models which are able to produce results similar to the real data remains as a task for future investigations.

### PageRank properties of various species

**Figure 6:**
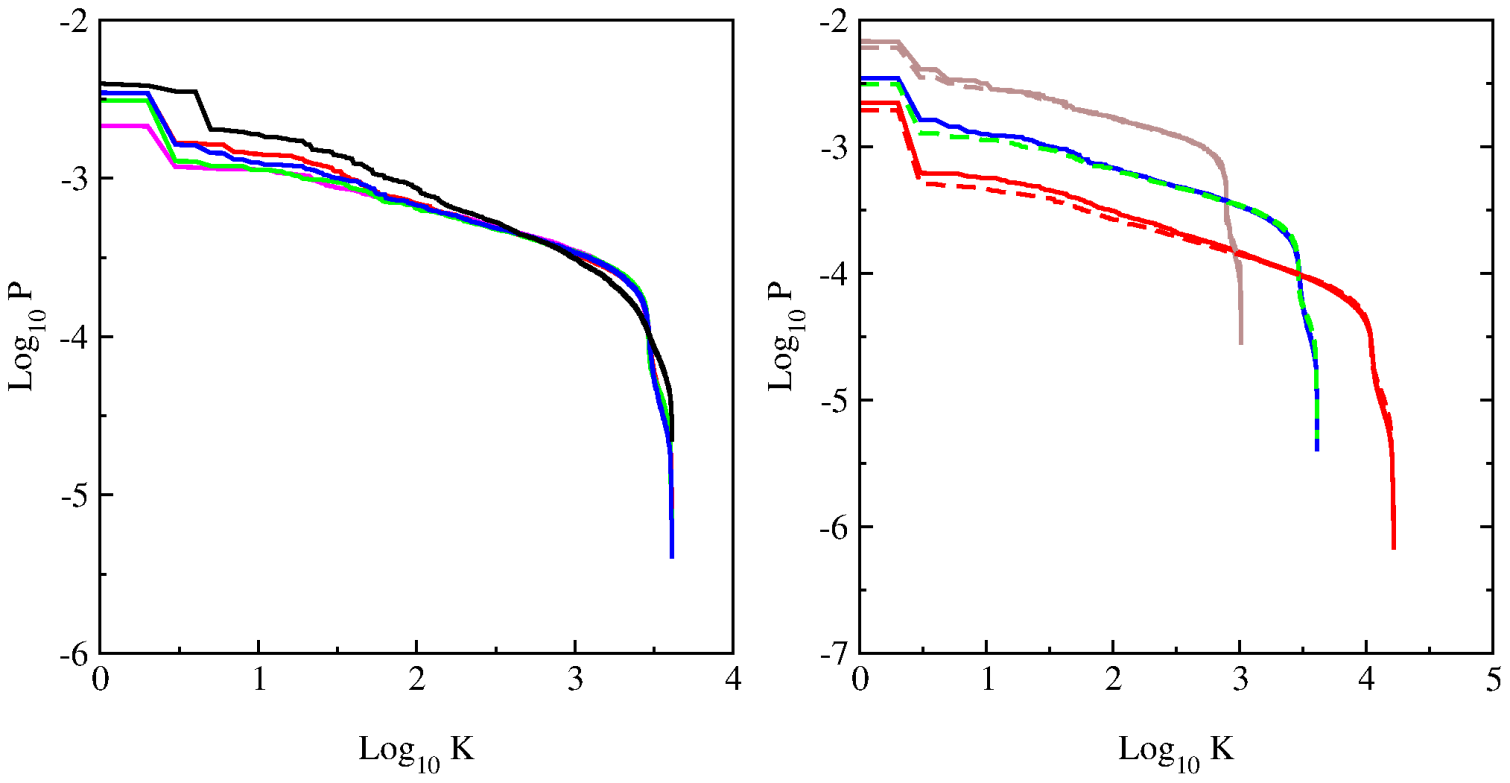
Dependence of PageRank probability 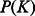 on PageRank index 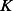. *Left panel :* Data for different species for word length of 6-letters: bull BT (magenta), dog CF (red), elephant LA (green), Homo sapiens HS (blue) and zebrafish DR (black). *Right panel :* Data for HS (full curve) and LA (dashed curve) for word length 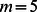 (brown), 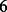 (blue/green), 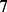 (red).

By numerical diagonalization of the Google matrix we determine the PageRank vector 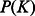 at 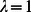 and several other eigenvectors with maximal values of 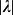. The dependence of probability 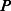 on index 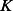 is shown in Fig. 6 for various species and different word length 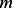. The probability 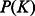 describes the steady state of random walks on the Markov chain and thus it gives the frequency of appearance of various words of length 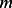 in the whole sequence 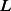. The frequencies or probabilities of words appearance in the sequences have been obtained in [13] by a direct counting of words along the sequence (the available sequences 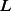 were shorted at that times). Both methods are mathematically equivalent and indeed our distributions 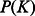 are in a good agreements with those found in [13] even if now we have a significantly better statistics.

The decay of 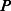 with 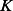 can be approximately described by a power law 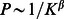. Thus for example for HS sequence at 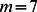 we find 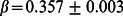 for the fit range 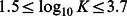 that is rather close to the exponent found in [13]. Since on average the PageRank probability is proportional to the number of ingoing links, or the sum of ingoing matrix elements of 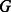, one has the relation between the exponent of PageRank 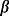 and exponent of ingoing links (or matrix elements): 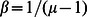 [3], [4], [23], [24], [25]. Indeed, for the HS DNA case at 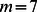 we have 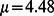 that gives 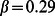 being close to the above value of 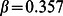 obtained from the direct fit of 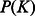 dependence. We think that the agreement is not so perfect since there is a visible curvature in the log-log plot of 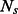 vs 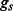 in Fig. 3. Also due to a small value of 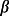 the variation range of 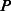 is not so large that reduces the accuracy of the numerical fit even if a formal statistical error is relatively small compared to a visible systematic nonlinear variations. In spite of this only approximate agreement we should say that in global the relation between 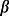 and 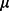 works correctly. In average we find for DNA network the value of 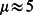 being significantly larger than for the WWW networks with 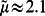 [3]. This gives a significantly smaller value 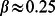 for DNA case comparing to the usual WWW value 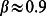 (we note that the randomized WWW networks and the Albert-Barabási model have 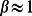 [26]). The relation between 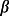 and 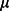 also works for the DR DNA case at 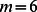 with 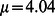 that gives 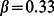 being in a satisfactory agreement with the fit value 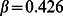 found from 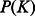 dependence of Fig. 6.

At 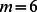 we find for our species the following values of exponent 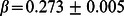 (BT), 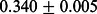 (CF), 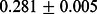 (LA), 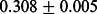 (HS), 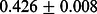 (DR) in the range 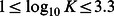. There is a relatively small variation of 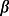 between various mammalian species. The data of Fig. 6 for HS show that the value of 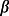 remains stable with the increase of word length. These observations are similar to those made in [13].

### PageRank proximity between species

**Figure 7:**
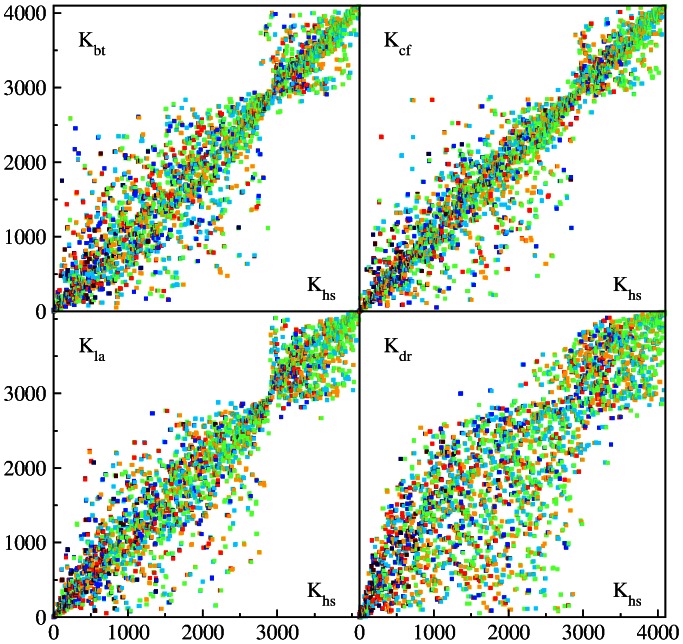
PageRank proximity 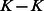 plane diagrams for different species in comparison with Homo sapiens: 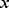-axis shows PageRank index 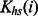 of a word 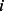 and 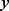-axis shows PageRank index of the same word 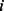 with 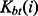 of bull, 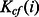 of dog, 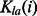 of elephant and 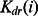 of zebrafish; here the word length is 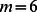. The colors of symbols marks the purine content in a word 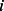 (fractions of letters 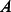 or 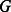 in any order); the color varies from red at maximal content, via brown, yellow, green, light blue, to blue at minimal zero content.

**Figure 8:**
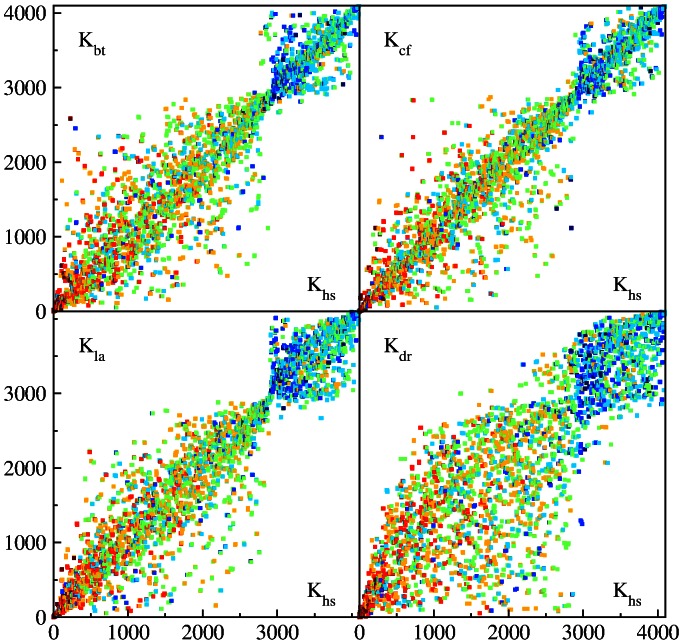
Same as in Fig. 7 but now the color marks the fraction of of letters 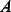 or 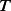 in any order in a word 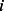 with red at maximal content and blue at zero content.

**Figure 9:**
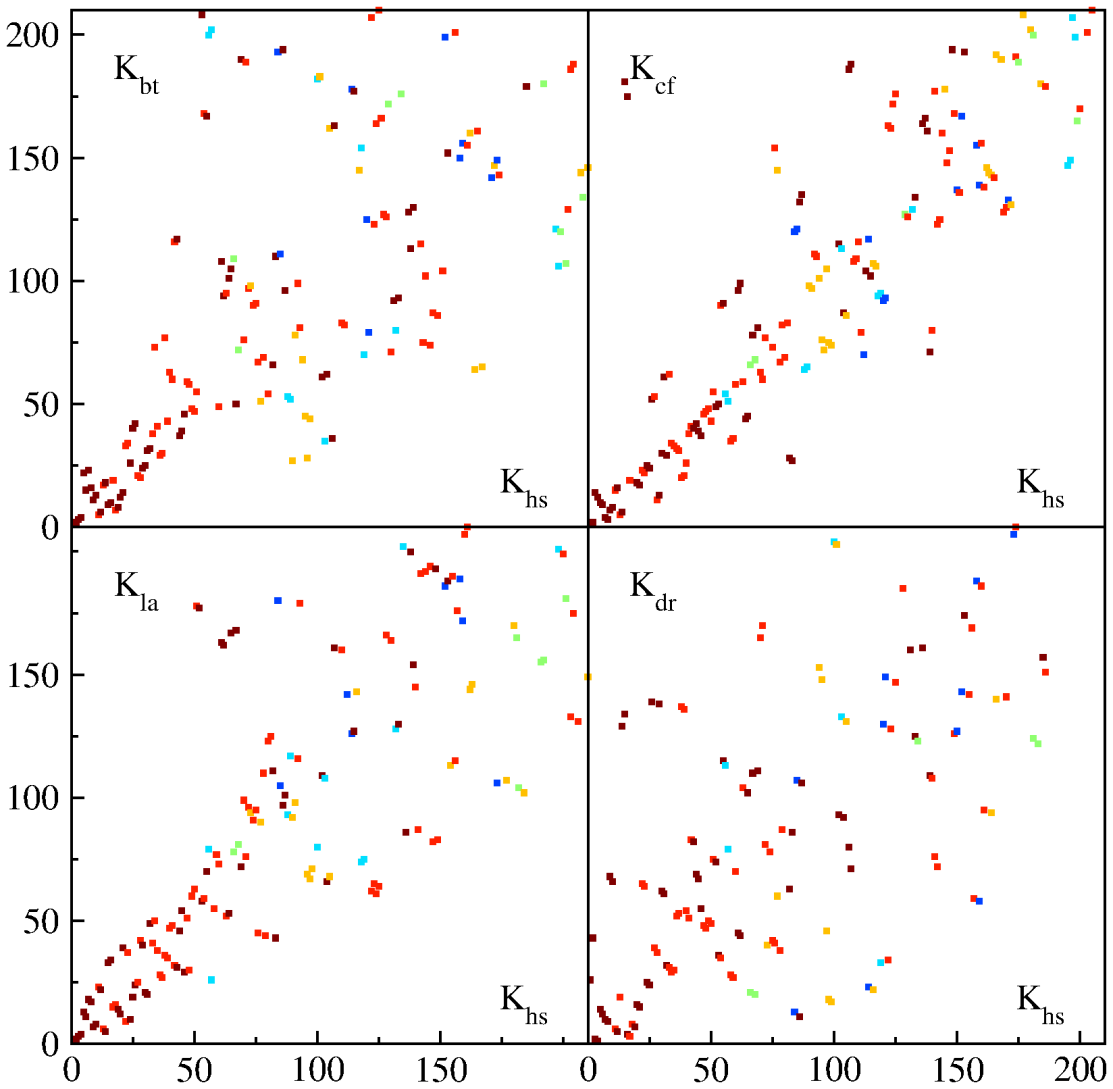
Zoom of the PageRank proximity 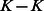 diagram of Fig. 8 for the range 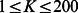 with the same color for 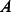 or 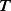 content.

The top ten 6-letters words, with largest probabilities 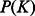, are given for all studied species in Table 1. Two top words are identical for BT, CF, HS. To see a similarity between species on a global scale it is convenient to plot the PageRank index 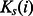 of a given species 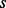 versus the index 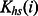 of HS for the same word 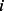. For identical sequences one should have all points on diagonal, while the deviations from diagonal characterize the differences between species. The examples of such PageRank proximity 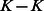 diagrams are shown in Figs. 7,8 for words at 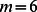. A zoom of data on a small scale at the range 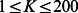 is shown in Fig. 9. A visual impression is that CF case has less deviations from HS rank compared to BT and LA. The non-mammalian DR case has most strong deviations from HS rank. For BT, CF and LA cases we have a significant reduction of deviations from diagonal around 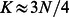. This effect is also visible for DR case even if being less pronounced. We do not have explanation for this observation.

**Table 1:** Top ten PageRank entries at DNA word length 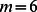 for species: bull BT, dog CF, elephant LA, Homo sapiens HS and zebrafish DR.

BT	CF	LA	HS	DR
TTTTTT	TTTTTT	AAAAAA	TTTTTT	ATATAT
AAAAAA	AAAAAA	TTTTTT	AAAAAA	TATATA
ATTTTT	AATAAA	ATTTTT	ATTTTT	AAAAAA
AAAAAT	TTTATT	AAAAAT	AAAAAT	TTTTTT
TTCTTT	AAATAA	AGAAAA	TATTTT	AATAAA
TTTTAA	TTATTT	TTTTCT	AAAATA	TTTATT
AAAGAA	AAAAAT	AAGAAA	TTTTTA	AAATAA
TTAAAA	ATTTTT	TTTCTT	TAAAAA	TTATTT
TTTTCT	TTTTTA	TTTTTA	TTATTT	CACACA
AGAAAA	TAAAAA	TAAAAA	AAATAA	TGTGTG

The fraction of purine letters 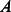 or 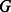 in a word of 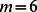 letters is shown by color in Fig. 7 for all words ranked by PageRank index 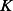. We see that these letters are approximately homogeneously distributed over the whole range of 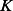 values. In contrast to that the distribution of letters 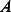 or 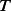 is inhomogeneous in 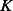: their fraction is dominant for 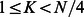, approximately homogeneous for 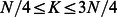 and is close to zero for 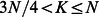 (see Fig. 8). We find that in the whole HS sequence the fractions 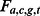 of 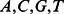 are respectively 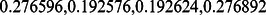 (and 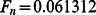 for undetermined 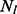). Thus we have the fraction of 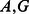 being close to 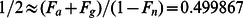 and the fraction of 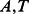 being 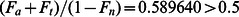. Thus it is more probable to have 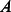 or 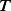 in the whole sequence that can be a possible origin of the inhomogeneous distribution of 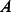 or 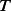 along 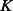 and large fraction of 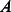, 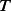 at top PageRank positions.

**Figure 10:**
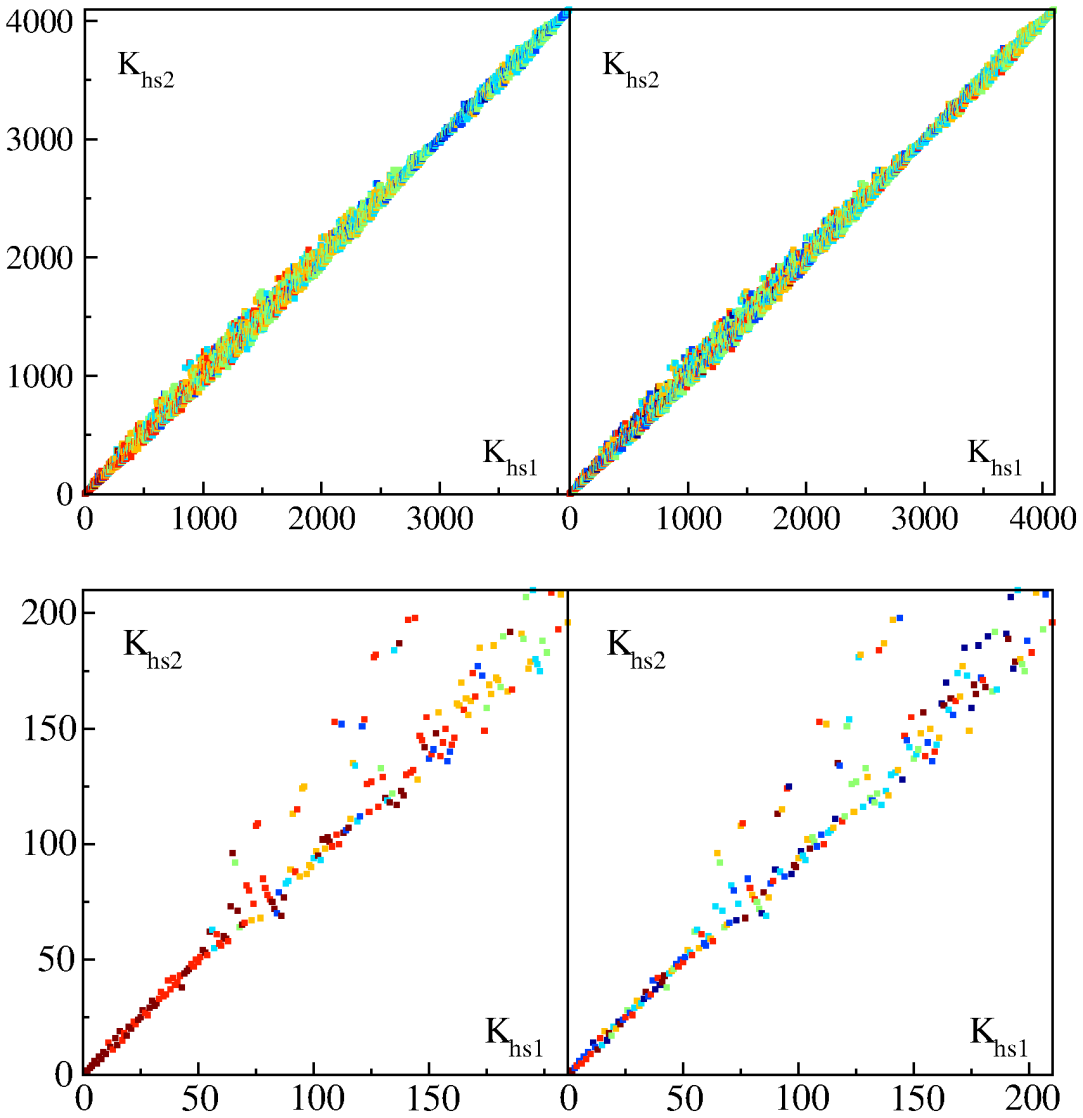
PageRank proximity 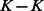 diagram of Homo sapiens 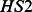 versus Homo sapiens 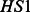 at 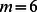 (see text for details). Top panels show the content of 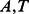 (left) and 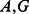 (right) in the same way as in Fig. 8 and Fig. 7 respectively. Bottom panels show zoom of top panels.

The whole HS sequence used here is composed from 5 humans with individual length 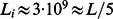. We consider the first and last fifth parts of the whole sequence 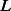 separately thus forming two independent sequences HS1 and HS2 of two individuals. We determine for the the corresponding PageRank indexes 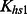 and 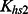 and show their PageRank proximity diagram in Fig. 10. In this case the points are much closer to diagonal compared to the case of comparison of HS with other species.

To characterize the proximity between different species or different HS individuals we compute the average dispersion 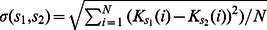 between two species (individuals) 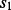 and 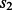. Comparing the words with length 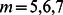 we find that the scaling 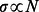 works with a good accuracy (about 10% when 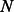 is increased by a factor 16). To represent the result in a form independent of 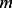 we compare the values of 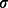 with the corresponding random model value 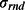. This value is computed assuming a random distribution of 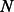 points in a square 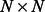 when only one point appears in each column and each line (e.g. at 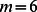 we have 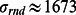 and 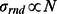). The dimensionless dispersion is then given by 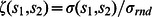. From the ranking of different species we obtain the following values at 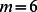: 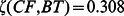; 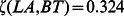, 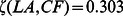; 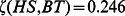, 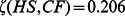, 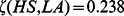; 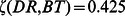, 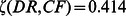, 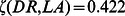, 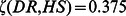 (other 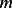 have similar values). According to this statistical analysis of PageRank proximity between species we find that 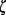 value is minimal between CF and HS showing that these are two most similar species among those considered here.

For two HS individuals we find 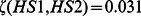 being significantly smaller then the proximity correlator between different species. We think that this PageRank proximity correlator 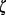 can be useful as a quantitative measure of statistical proximity between various species.

Finally, in Table 2 we give for all species the words of 6 letters with the 10 minimal PageRank probabilities. Thus for HS the less probable is the word TACGCG corresponding to two amino acids Tyr and Ala. In general the ten last words are mainly composed of C and G even if the letters A and T still have small but nonzero weight. The last two words are the same for mammalian species but they are different for DR sequence.

**Table 2:** Ten words with minimal PageRank probability given at 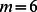 for species: bull BT, dog CF, elephant LA, Homo sapiens HS and zebrafish DR.

BT	CF	LA	HS	DR
CGCGTA	TACGCG	CGCGTA	TACGCG	CCGACG
TACGCG	CGCGTA	TACGCG	CGCGTA	CGTCGG
CGTACG	TCGCGA	ATCGCG	CGTACG	CGTCGA
CGATCG	CGTACG	TCGCGA	TCGACG	TCGACG
ATCGCG	CGATCG	CGCGAT	CGTCGA	TCGTCG
CGCGAT	CGAACG	GTCGCG	CGATCG	CCGTCG
TCGACG	CGTTCG	CGATCG	CGTTCG	CGACGG
CGTCGA	TCGACG	CGCGAC	CGAACG	CGACCG
CGTTCG	CGTCGA	TCGCGC	CGACGA	CGGTCG
TCGTCG	ACGCGA	ACGCGA	CGCGAA	CGACGA

Here the top row is the last PageRank entry, bottom is the tenth one from the end of PageRank.

### Other eigenvectors of G

**Figure 11:**
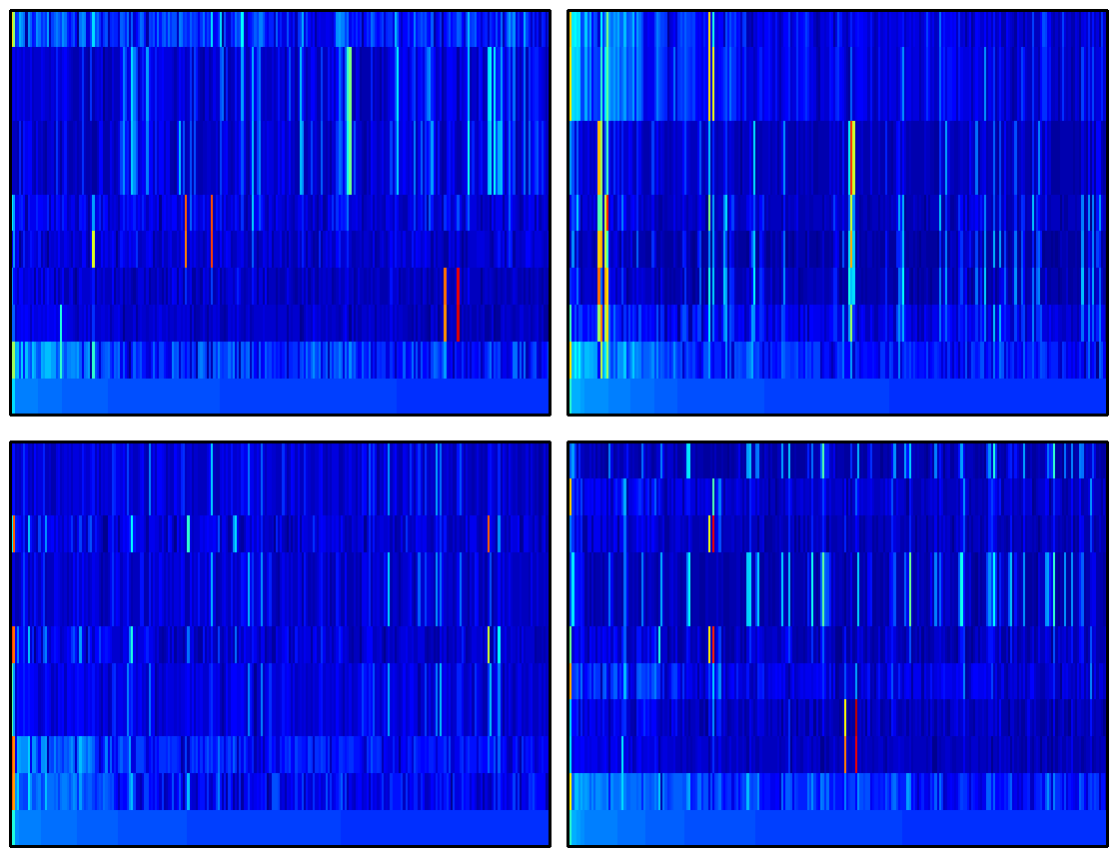
Dependence of eigenstates amplitude 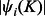 on PageRank index 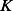 in 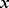-axis and eigenvalue index 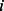 in 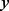-axis for largest ten eigenvalues 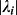 counted by 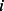 from 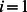 at 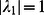 to 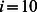 at 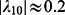. The range 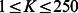 is shown with PageRank vector for a given species at the bottom line of each panel. For each species in each panel the color is proportional to 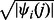 changing from blue at zero to red at maximal amplitude value which is close to unity in each panel. The panels show the species: bull BT (top left), dog CF (top right), elephant LA (bottom left), Homo sapiens HS (bottom right).

The properties of 10 eigenstates 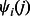 of DNA Google matrix with largest modulus of eigenvalues 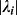 are analyzed in Table 3 and Fig. 11. The words 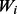 at the maximal amplitude 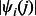 are presented for all species in Table 3. We see that in general these words 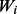 are rather different from the top PageRank word 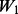 (some words appear in pairs since there are pairs of complex conjugated values 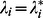).

**Table 3:** Words 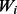 corresponding to the maximum value of eigenvector modulus 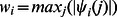 for species bull BT, dog CF, elephant LA, Homo sapiens HS and zebrafish DR, which are shown in dark red in Fig. 11.

i	BT	CF	LA	HS	DR
1	TTTTTT	TTTTTT	AAAAAA	TTTTTT	ATATAT
2	TTTTTT	AAAAAA	AAAAAA	TTTTTT	TATATA
3	ACACAC	CTCTCT	AAAAAA	ACACAC	ATATAT
4	ACACAC	AGAGAG	AAAAAA	ACACAC	TAGATA
5	CACACA	CTCTCT	AAAAAA	TTTTTT	ATAGAT
6	CACACA	TCTCTC	AAAAAA	CACACA	TATCTA
7	CCAGGC	AGAGAG	TATGAG	TGGGAG	ATCTAT
8	CCAGGC	AGAGAG	TATGAG	TGGGAG	TAGATA
9	CCCATG	TGTGTG	TTTTTT	CACACA	ATAGAT
10	CCCATG	TGTGTG	AGAGTA	TTTTTT	TATCTA

The eigenvectors at 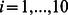 correspond to the ten largest eigenvalues 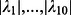 of the DNA Google matrix for DNA word length 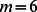. The first row 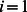 corresponds to top PageRank entries.

The probability of the above top 10 eigenstates as a function of PageRank index 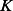 are shown in Fig. 11. We see that the majority of the vectors, different from the PageRank vector, have well localized peaks at relatively large values 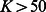. This shows that in the DNA network there are some modes located on certain specific patterns of words.

To illustrated the localized structure of eigenmodes 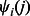 for HS case at 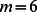 we compute the inverse participation ratio 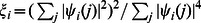 which gives an approximate number of nodes on which the main probability of an eigenstate 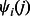 is located (see e.g. [4], [21], [26]). The obtained values are 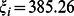, 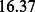, 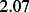, 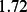, 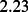, 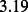, 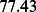, 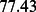, 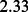, 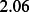 for 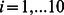 respectively. We see that for 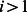 we have significantly smaller 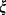 values compared to the case of PageRank vector with a large 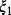. This supports the conclusion about localized structure of a large fraction of eigenvectors of 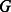.

In [22] on an example of Wikipedia network it is shown that the eigenstates with relatively large 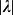 select specific communities of the network. The detection of communities in complex networks is now an active research direction [27]. We expect that the eigenmodes of G matrix can select specific words of bioniformatic interest. However, a detailed analysis of words from eigenmodes remains for further more detailed investigations.

## Discussion

In this work we used long DNA sequences of various species to construct from them the Markov process describing the probabilistic transitions between words of up to 7 letters length. We construct the Google matrix of such transitions with the size up to 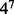 and analyze the statistical properties of its matrix elements. We show that for all 5 species, studied in this work, the matrix elements of significant amplitude have a power law distribution with the exponent 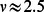 being close to the exponent of outgoing links distribution typical for WWW and other complex directed networks with 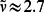. The distribution of significant values of the sum of ingoing matrix elements of 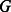 is also described by a power law with the exponent 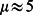 which is significantly larger than the corresponding exponent for WWW networks with 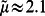. We show that similar to the WWW networks the exponent 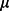 determines the exponent 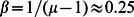 of the algebraic PageRank decay which is significantly smaller then its value for WWW networks with 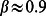. The PageRank decay is similar to the frequency decay of various words studied previously in [13]. It is interesting to note that the value 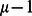 is close to the exponent of Poincaré recurrences decay which has a value close to 4 [12] (even if we cannot derive a direct mathematical relation between them).

Using PageRank vectors of various species we introduce the PageRank proximity correlator 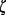 which allows to measure in a quantitative way the proximity between different species. This parameter remains stable in respect to variation of the word length.

The spectrum of the Google matrix is determined and it is shown that it is characterized by a significant gap between 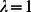 and other eigenvalues. Thus, this spectrum is qualitatively different from the WWW case where the gap is absent at the damping factor 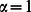. We show that the eigenmodes with largest values of 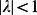 are well localized on specific words and we argue that the words corresponding to such localized modes can play an interesting role in bioinformatic properties of DNA sequences.

Finally we would like to trace parallels between the Google matrix analysis of words in DNA sequences and the small world properties of human language. Indeed, it is known that the frequency of words in natural languages follows a power law Zipf distribution with the exponent 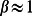 [28]. The parallels between words distributions in DNA sequences and statistical linguistics were already pointed in [13]. The analysis of degree distributions of undirected networks of words in natural languages was found to follow a power law with an exponent 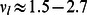 [29] being not so far from the one found here for the matrix elements distribution. It is argued that the language evolution plays an important role in the formation of such a distribution in languages [30]. The parallels between linguistics and DNA sequence complexity are actively discussed in bioinformatics [31], [32]. We think that the Google matrix analysis can provide new insights in the construction and characterization of information flows on DNA sequence networks extending recent steps done in [33].

In summary, our results show that the distributions of significant matrix elements are similar to those of the scale-free type networks like WWW, Wikipedia and linguistic networks. In analogy with lingusitic networks it can be useful to go from words network analysis to a more advanced functional level of links inside sentences that may be viewed as a network of links between amino acids or more complex biological constructions.

## Supporting Information

Supporting Information S1 Supplementary methods, references, tables, sequences data and figures are available at: http://www.quantware.ups-tlse.fr/QWLIB/dnagooglematrix/.
(TXT)
